# Lacrimal Gland Involvement in Lymphomatoid Granulomatosis and Review of the Literature

**DOI:** 10.1155/2010/358121

**Published:** 2010-09-01

**Authors:** Thanuja Gopal Pradeep, Paul Cannon, Thomas Dodd, Dinesh Selva

**Affiliations:** ^1^South Australian Institute of Ophthalmology and Discipline of Ophthalmology and Visual Sciences, University of Adelaide, Adelaide, SA 5000, Australia; ^2^Department of Ophthalmology and Visual Sciences, Royal Adelaide Hospital, North Terrace, Adelaide, SA 5000, Australia; ^3^Institute of Medical and Veterinary Sciences, University of Adelaide, Adelaide, SA 5000, Australia

## Abstract

*Objective*. To describe the clinicoradiological and histopathological findings in a case of lacrimal gland enlargement secondary to lymphomatoid granulomatosis (LG) and to review the literature. *Design*. Case report and systematic literature review. *Methods*. A 75-year-old woman presented with right ptosis. Computerised tomography showed lacrimal gland enlargement, and biopsy done was inconclusive. She subsequently developed pulmonary symptoms and underwent transbronchial biopsy that was diagnosed as LG. Pub Med and OVID databases were searched using the term “orbit/eye involvement in lymphomatoid granulomatosis”. Articles that predated the databases were gathered from current references. *Results*. The patient underwent lacrimal gland biopsy which revealed necrotic and inflamed tissue with no further categorisation but transbronchial biopsy helped in establishing the diagnosis of LG. On initiation of prednisolone and cyclophosphamide, her orbital lesion resolved but the patient died following massive pulmonary hemorrhage within a month of diagnosis. *Conclusion*. Ophthalmic involvement in LG is very rare. Varied presentations are due to central nervous system involvement, vasculitis, or infiltration of ocular or orbital structures. LG is an angiocentric and angiodestructive granulomatous disorder and can involve any tissue, thus accounting for the variable presentations reported in literature.

## 1. Introduction

LG is an Epstein-Barr virus- (EBV-) associated angiocentric B-cell lymphoproliferative process. The lung is the most commonly involved organ, but skin, central nervous system, kidney, and gastrointestinal tract can also be involved. There are few reports of ophthalmic involvement in LG. We report a patient with LG who presented with dacryoadenitis and review the literature on the ophthalmic manifestations of this condition.

## 2. Case Report

**Figure 1:**
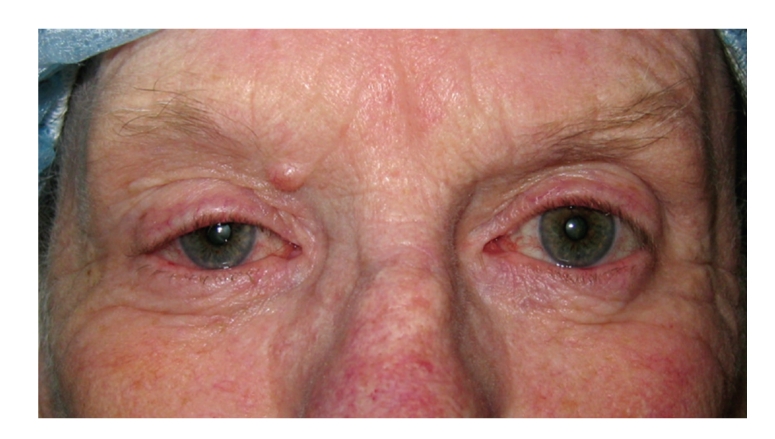
Clinical photograph showing inferomedial displacement of the globe.

**Figure 2:**
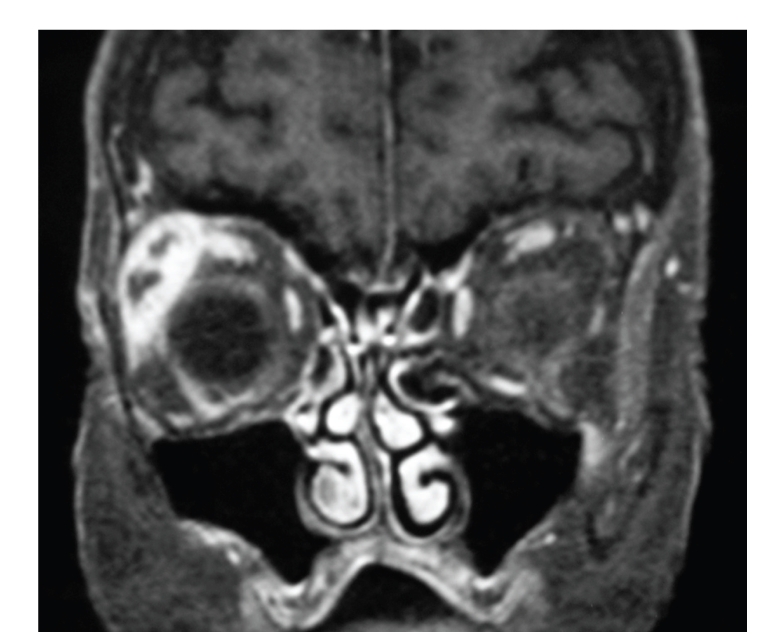
T1 weighted contrast enhanced MRI scan coronal sections through the orbits showing contrast enhancing lacrimal gland lesions with central hypodense areas.

**Figure 3:**
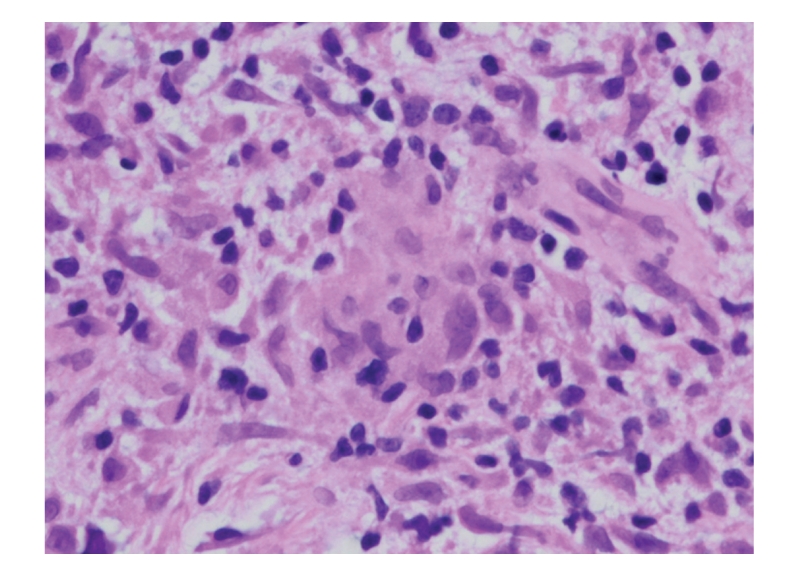
Photomicrograph of lacrimal gland biopsy shows a lympho-histiocytic infiltrate with reactive fibroblasts (hematoxylin-eosin, original magnification ×400).

A 75-year-old woman developed a painless acute right ptosis associated with double vision and vertigo. Examination revealed inferomedial globe displacement and an S-shaped ptosis (Figure 1). Computerized tomography (CT) revealed an enlarged right lacrimal gland with areas of central hypodensity (Figure 2). The medical history included Sjogren's syndrome, abdominal aortic aneurysm, and colonic adenocarcinoma 2 years previously that was treated surgically. The lacrimal gland was biopsied through a superior lid crease approach. Histopathology was inconclusive showing only necrotic and inflamed tissue with granulomatous inflammation (Figure 3). A further biopsy was declined by the patient.

She subsequently developed fever, malaise, and cough. A CT of the chest showed a necrotic mass involving her left upper lobe and hilar vessels with a suspicious right lung lesion. Hilar and mediastinal lymphadenopathy was noted. CT abdomen revealed multiple hepatic lesions. She underwent bronchial biopsy and the histopathology demonstrated florid necrotizing inflammation with palisading granulomas and a probable diagnosis of Wegner's granulomatosis was made. Her blood investigations showed elevated CRP levels (150 mg/l) and anemia (Hb-10 g/dl). ANCA was negative and CEA levels were normal (2 *μ*g/L). The patient was started on Prednisolone 25 mg/day and Cyclophosphamide 100 mg/day. 

**Figure 4:**
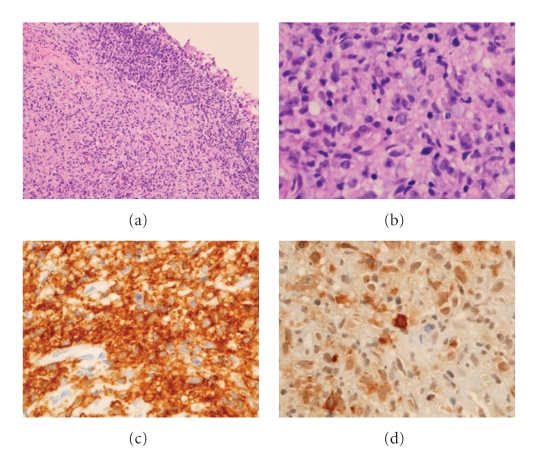
Transbronchial biopsy. (a) Photomicrograph of the transbronchial biopsy demonstrates submucosal lymphocytic inflammatory infiltrate (hematoxylin-eosin, original magnification ×100). (b) Higher magnification shows a polymorphous lymphoid population with atypical lymphoid cells (hematoxylin-eosin, original magnifiaction ×400). (c) Immunohistochemistry illustrates that the cells are predominantly B-cells (CD20 immunohistochemical stain, original magnification ×200). (d) Immunohistochemistry for Epstein-Barr virus antigen demonstrates positive staining (EBV-LMP immunohistochemical stain, original magnification ×400).

She was readmitted 1 month later with further deterioration of her systemic condition. CT of the orbits were normal with complete resolution of the lacrimal gland enlargement. However, her CT of the chest revealed development of left lower lobe parenchymal infiltrates and resolution of liver lesions when compared with the previous study. She underwent a repeat bronchial biopsy and the histopathology showed a polymorphous atypical lymphoid infiltrate of CD20 positive B cells with positive labelling for EBV admixed with CD3 positive T cells. The lesion displayed focal necrosis and focal angiocentricity and a diagnosis of LG with areas of high grade involvement was made (Figure 4). Microbiology yielded Staphylococcus aureus. She was treated with antibiotics and steroids and cyclophosphamide. She underwent bone marrow biopsy which showed reactive lymphoid aggregates but ruled out lymphoma. Patient continued to deteriorate and died 1 month later due to massive pulmonary hemorrhage.

## 3. Discussion

LG was first described by Leibow et al. [1] in 1972 as an atypical angiocentric and angiodestructive lymphoproliferative disease involving lungs as well as other extra nodal sites, mainly skin, CNS, and kidneys. In recent years it has been shown to be a T-cell rich EBV-related lymphoproliferative disease [17]. It can present at any age although 80% of the cases occur between 4th and 6th decades. The incidence is higher in men with a male-to-female ratio of 1.7 : 1 [3]. As with other EBV-associated lymphoproliferative disorders (such as post-transplant lymphoproliferative disorder), LG occurs with increased frequency in immunosuppressed patients. In its most indolent forms, it presents with pulmonary/skin nodules in an otherwise asymptomatic patient that resolve without any treatment. Fewer than 20% of the patients present with eye involvement, hepatosplenomegaly, adenopathy, and arthralgia [18]. Around 14–27% of the patients achieve durable remissions without treatment though a more likely course is that of relentless progression with multiple organ involvement and progression to large-cell lymphoma with a median survival of 14 months [3]. 

LG is characterized histologically by a polymorphic lymphoid infiltrate consisting of mature lymphocytes, eosinophils, histiocytes, and atypical lymphoreticular cells [1]. Focal areas of necrosis and transmural inflammation of vessels (angiitis) are also typical and extensive necrosis and monomorphic large-cell lymphoid infiltrate are indicative of a high-grade lesion. Since the histological findings are highly reminiscent of Wegener's granulomatosis, a much more common condition, the diagnosis of LG needs to be confirmed by demonstrating the presence of EBV positive B-cells within the lesion. A 3-tier grading system has been proposed based on the number of EBV positive cells [19]. The histological grading may vary over time or from site to site [18] and hence multiple site or repeated biopsies are essential to establish the diagnosis in cases with high index of clinical suspicion. 

 No well-studied effective treatment exists for this disease. Traditional treatment regimens were centered on immunosuppression (with cyclophosphamide and prednisolone) [5] but since the recognition that LGY is an EBV driven disease, antiviral therapy in the form of interferon-alpha is increasingly employed [18]. Combination chemotherapy is reserved for high-grade lesions. Newer therapies such as rituximab and autologous stem cell transplantation have also given good results [19–21]. Radiotherapy has also been used in certain cases [2], and proven to be an effective adjunct in treating localized disease.

 The reports of ophthalmic involvement in LG [1–16, 22, 23] have been summarized in Table 1. Twenty one cases were reviewed. Four cases were excluded because sufficient information was not available [3, 5]. Excluding these cases, details of the remaining 17 cases were obtained and the age of onset, sex, ophthalmic presentation, the tissue biopsy used to establish diagnosis, treatment, and outcome were assessed. 

The age at presentation ranged from 7 to 75 years (mean: 43.9 years) with equal incidence in both sexes: (9 male : 9 females). Of the 18 cases including our case, six presented with ocular symptoms while the remainder had pulmonary symptoms or central nervous involvement at initial diagnosis. The ophthalmic manifestations were due to infiltration of the ocular and orbital tissues in 12 cases; vasculitis caused functional disturbances in 3 cases; and ophthalmic expressions of the disease were seen in 4 cases from CNS disease. Eight of the 18 cases (44.4%) presented acutely with progressive development of ocular manifestations within a month. The diagnosis was established in most cases by lung biopsy (13 cases). Only 4 cases had ophthalmic manifestations secondary to central nervous system (CNS) involvement (including diplopia, ptosis, nystagmus, and tonic pupil) contrary to earlier reports that ocular involvement in LG is commonly due to CNS involvement [23]. 

 Five cases were treated with radiotherapy for the ocular manifestations. These included orbital infiltrations, eyelid swelling, and one case of conjunctival involvement, with all cases showing very good response with regression of symptoms. The remaining cases were treated with corticosteroids and/or immunosuppressants and in all cases the ocular manifestations resolved partially or completely except cases of optic neuropathy and optic nerve infarction. The follow up period for the cases ranged from 6 weeks to twelve years (mean = 3.15 years). Of the 17 cases where follow up information was available, 8 succumbed to the disease with a median survival of 6 months (range: 6 weeks to 2 years). 

 In our case, the CT of the orbit revealed a nonhomogenous enlargement of lacrimal gland with central hypodense areas which corresponded with areas of necrosis within the lacrimal gland. This feature was of notable significance as the differential diagnoses included very few conditions such as necrotic or cystic changes within a pleomorphic or mucoepidermoid tumor of lacrimal gland and acute dacryoadenitis with abscesses within the gland [24–26].

Though ophthalmic manifestations are uncommon, they may rarely be the presenting feature in LG. Ophthalmologists should be aware of this condition, especially since many of the manifestations can mimic those of Wegener's granulomatosis. It is also a condition which may require multiple tissue biopsies to establish the diagnosis and one should not hesitate to do so in cases with high index of clinical suspicion. Given the systemic nature of the disease and poor prognosis of late-stage high-grade lesions, early diagnosis and appropriate treatment by an expert with an interest in this condition may offer the best hope for the patient.

## Figures and Tables

**Table 1:** Lymphomatoid granulomatosis: clinical data.

Study	Age/Sex	Ocular manifestations	Onset	Extraocular manifestations	LYG established by biopsy	Initial presentation	Treatment	Follow up	Outcome
Leibow et al. [1]	33/M	Periorbital swelling	Acute	Sialadenitis		Sialadenitis and peri orbital swelling		1 1/2 months	D

	8 1/2/F	Ptosis,bilateral nystagmus,pale optic disc	Acute (2weeks)	CNS involvement	Wedge biopsy of lung	Systemic sym ptoms, ocular and CNS	Prednisolone	3 years	A

Shank et al. [2]	62/F	Supra orbital swelling	Acute (4 weeks)	lymph nodes, lung, CML	Open lung biopsy	Erythema nodosum	Radiotherapy, prednisolone, vincristine, cyclophosphamide	1 year	D

Katzenstein et al. [3]	NA	Atypical lyphoma of eyelids	NA	NA	NA	NA			

	NA	Uveitis	NA	NA	NA	NA			

Sordillo et al. [4]	62/M	Unilateral proptosis, lacrimal gland, conjunctival involvement	NA	Adenopathy pulmonary	Lymph node, lung	proptosis	Radiotherapy prednisolone, chemotherapy	4 years	A

	60/F	Orbital mass, choroidal infilt, CRVO	NA	Adenopathy, fever, pulmonary	Lung	Lymphadenopathy	Radiothaerpy, prednisolone, vincristine, cyclophosphamide	2 years	D

Fauci et al. [5]	NA		NA	NA	NA				

	NA		NA	NA	NA				

Saraux et al. [6]	35/F	Retinal arteritis, ptosis mydriasis	NA		Open lung biopsy	Rhinopharyngitis, pneumenia	Corticosteroids, cyclophosphamide, plasmapheresis	6 months	D

Tse et al. [7]	29/M	Bilateral peripheral retinal vasculitis and uveitis, decreased lacrimation	Chronic (3 months)	Pulmonary, cerebellar	open lung biopsy	rhinorrhea, dry cough, malaise	Prednisolone, radiotherapy	6 months	D

Chung YM et al. [8]	62/F	Ulcerative conjunctival nodule	NA		Lung biopsy			NA	

Kinyoun et al. [9]	56/M	Inflammation of choriocapillaris	Chronic (6 months)	Pulmonary	Open lung biosy	productive cough	Corticosteroids, cyclophosphamide	12 years	A

Font et al. [10]	62/M	Eyelid and eyebrow involvement	Chronic (4 months)	Skin, pulmonary, adenopathy	Skin biopsy from eyebrow	skin lesions	Antibiotics, chemotherapy	3 years	D

McKay et al. [11]	60/F	Retrobulbar optic nerve infarction, Scleritis, ? Orbital infiltration	Acute		Nasal mucosa, sclera	presented with bilateral red eye	Radiotherapy, steroid	2 years	A

Pearson ADJ et al. [12]	13/M	Bilateral choroidal infiltration, diplopia sec to CN inv	NA	Pulmonary	Open lung biopsy	respiratory failure	Vincristine, cyclophosphamide, prednisolone	6 years	A

Haider. [13]	31/M	Tonic pupil	Acute (3 weeks)	GIT, skin	Skin	abdominal pain		NA	A

Moertel et al. [14]	7/F	Tonic pupil	NA	ALL, cerebral nodule, pulmonary	Lung biopsy	Lymphadenopathy, splenomegaly, pulmonary Infiltrates	Chemotherapy, dexamethasone	7 years	A

Forman et al. [14]	41/F	Unilateral optic neuropathy	Acute (1 week)	Pulmonary	Lung biopsy	monoocular visual loss		2 months	D

Head et al. [15]	54/M	Conjunctiavl growth	Sub acute Several wks	NA	Open lung biopsy	pulmonary	Radiotherapy to orbit	NA	A

Cameron and Cackett et al. [16]	39/M	Bilateral exudative retinal detachment	Acute (3 days)	Pulmonary, skin	Skin	cough, dyspnoea skin rash	Prednisolone	2 years	A

Pradeep et al.	75/F	Unilateral dacryoadenitis	Acute (2 weeks)	Pulmonary	Transbronchial biopsy	dacryoadenitis	Prednisolone and cyclophosphamide	6 months	D

F: female, M: male; NA-not available; CNS- central nervous system; GIT-gastrointestinal tract; ALL: Acute lymphocytic leukemia; CML- chronic myeloid leukemia; A: alive at the end of follow up period; D dead at the end of follow up period.
